# Destabilising Effect of Class B CpG Adjuvants on Different Proteins and Vaccine Candidates

**DOI:** 10.3390/vaccines13040395

**Published:** 2025-04-08

**Authors:** Kawkab Kanjo, Rakesh Lothe, Gaurav Nagar, Meghraj Rajurkar, Harish Rao, Saurabh Batwal, Umesh Shaligram, Raghavan Varadarajan

**Affiliations:** 1 Molecular Biophysics Unit, Indian Institute of Science, Bangalore 560012, India; kawkabkanjo@iisc.ac.in; 2 Serum Institute of India Pvt. Ltd., Pune 411028, India; rakesh.lothe@seruminstitute.com (R.L.); gaurav.nagar@seruminstitute.com (G.N.); meghraj.rajurkar@seruminstitute.com (M.R.); harish.rao@seruminstitute.com (H.R.); saurabh.batwal@seruminstitute.com (S.B.); umesh.shaligram@seruminstitute.com (U.S.)

**Keywords:** CpG, SARS-CoV-2 RBD, stability, adjuvant, vaccine, proteolytic sensitivity, thermal stability

## Abstract

**Background:** Adjuvants function by enhancing the breadth, durability, and magnitude of the immune response, but little is known about their impact on vaccine stability. CpG is a widely used adjuvant that is included in several recently approved COVID-19 vaccines using Spike protein, RBD, or whole inactivated virus. **Methods**: Here, we investigate the in vitro stability of the Receptor-Binding Domain (RBD) of the SARS-CoV-2 Spike protein, as well as a number of other proteins formulated with a class B CpG adjuvant. **Results**: We show that RBD, BSA, and lysozyme proteins are less thermally stable, more aggregation-prone, and more protease-sensitive in the presence of CpG than without it, and that these effects are enhanced with prolonged incubation. For RBD, the effects of CpG are pH-independent but dependent on the salt concentration, with relative destabilisation decreasing with an increasing salt concentration, indicative of an electrostatic component to the interaction between CpG and the protein. The reduced thermal and proteolytic stability found in the presence of CpG is indicative of a preferential interaction of CpG with the unfolded state of the protein relative to its native state. It remains to be determined if these in vitro characteristics are unique to CpG or are also shared by other non-CpG commercial adjuvants, if they are antigen-dependent, and if and how they correlate with the in vivo immunogenicity of an adjuvanted vaccine. **Conclusions**: It is demonstrated that the CpG adjuvant is critical to enhancing immunogenicity and is a key reason for the success of multiple licensed commercial vaccines. Nonetheless, our work suggests that careful and systematic in vitro formulation studies may be warranted for the development of suitable, stable formulations of CpG-adjuvanted vaccine candidates.

## 1. Introduction

Adjuvants are added to vaccine formulations to enhance the immune response and increase its breadth and durability [1]. A wide range of adjuvants have been developed, but only very few are approved for human clinical use in various vaccine formulations [2]. Cytosine-phosphoguanosine oligodeoxynucleotide (CpG ODN) adjuvants are nucleic acid-based adjuvants containing CpG unmethylated dinucleotides [2]. CpG-containing ODNs (hereafter referred to as CpG for conciseness) are TLR9 ligands that work by augmenting B cell responses and inducing the maturation of plasmacytoid dendritic cells, as well as inducing potent IFN-α responses and promoting Th1 cellular responses [3,4,5]. CpG derivatives are considered to be an attractive choice of adjuvant. CpG improves immunogenicity, is used in licenced vaccines, including the Hepatitis B virus (HBV) vaccine, and is well tolerated with an established safety profile and efficacy in humans and animals [6,7,8]. The inclusion of CpG as an adjuvant in vaccines has been proven to enhance immunogenicity and augment the CD4+ T cell response in elderly mice [9,10] and restore defects in Th1 responses in aged pigs [11] and aged mice [12,13,14]. In the context of an HBV vaccine, CpG ODN was safe and enhanced seroprotection against HBV in human adults across multiple age groups [15,16]. It has been approved for human use in COVID-19 in clinical trials involving adults and older human populations [17,18], and recently, a vaccine formulation (CORBEVAX) comprising the Receptor-Binding Domain (RBD) of the spike protein of SARS-CoV-2 formulated with CpG was also given Emergency Use Listing (EUL) in India and Botswana [19,20].

Based on their structure and the immune response they induce, CpG adjuvants are classified into four distinct classes. Class A ODNs (D-type) consist of a phosphodiester core and a flanking region of phosphorothioate nucleotides. This ODN class harbours a single CpG motif flanked by palindromic sequences, which allow for the formation of a stem-loop structure. Class A ODNs function by inducing IFN-γ secretion from Natural Killer (NK) cells and maturation and IFN-α secretion from plasmacytoid dendritic cells (PDCs), without any effect on B cells [21,22]. Class B, or K-type, oligodinucleotides (ODNs) encode multiple (1–5) CpG motifs on a phosphorothioate backbone, helping to prolong the half-life of the ODN and rendering it DNase-resistant [23]. The ODNs in this class stimulate the proliferation and IgM secretion of B cells and trigger PDC differentiation and modest IFN-α production [22,24]. Class C ODNs have a phosphorothioate backbone and carry multiple palindromic CpG motifs that can form stem-loop structures. This CpG class stimulates the proliferation and differentiation of B cells and PDCs and triggers IL-6 and IFN-α production [25,26]. The fourth class of CpG adjuvants is class P, whose structure contains a phosphorothioate backbone, multiple CpG motifs, and two palindromes. Class P ODNs induce the strongest type I IFN secretion [27,28]. Despite their common use in formulations for a wide range of vaccines, the effect of CpG adjuvants on vaccine durability and stability has not been extensively studied. CpG adjuvants, most commonly class B, have been used in vaccine formulations as standalone adjuvants or in combination with alum [14,29,30,31,32,33]. In this study, we investigated the stability of different proteins and vaccine candidates alone or formulated with two different class B CpG adjuvants. In our in vitro study, CpG displayed a destabilising effect on RBD, BSA, and lysozyme proteins, and a neutral effect on others. This destabilising effect manifested as reduced thermal stability, increased proteolytic sensitivity, and reduced conformational stability, especially with prolonged incubation.

## 2. Materials and Methods

### 2.1. Reagents

CpG 1018^®^ Adjuvant (Dynavax Technologies, Emeryville, CA, USA) is the adjuvant in the FDA-approved Hepatitis B vaccine HEPLISAV-B^®^ (Dynavax Technologies, Emeryville, CA, USA) and RBD–Hepatitis B surface antigen conjugate (RBD–HBsAg were provided by the Serum Institute of India (SII), Pune, India). CpG7909 was a kind gift from Mynvax Private Limited, Bengaluru, India.

### 2.2. Recombinant Protein Expression and Purification

RBD proteins and RBD–Hepatitis B Surface Antigen (HBsAg) conjugates were prepared from proteins expressed in *Pichia pastoris* and purified at the Serum Institute of India (SII), as described previously [34]. Spy-Catcher and Spy-Tag, i.e., plug-and-display technology, were used to present the antigen of RBD SARS CoV-2 on HBsAg VLPs. The quality and quantity of the immune response can be efficiently improved by presenting antigens to the immune system on virus-like particles (VLPs) [35]. VLPs are non-infective and non-replicating, since they are essentially devoid of infectious genetic material. VLPs can display antigenic epitopes in the correct conformation and in a highly repetitive manner, leading to the cross-linking of B cell immunoglobulin receptors and B cell activation [36]. These strategies have shown a good efficacy in inducing potent immune responses in the host, characterised by efficient B and T cell responses, as well as long-lived immunity [37]. Various techniques are available to pair VLPs with the antigen(s) of interest, including genetic fusion, chemical derivatisation, conjugation, or “plug-and-display” decoration, which forms a spontaneous iso-peptide bond between a peptide and its protein conjugate derived from a specific domain of a particular bacterial protein [38,39]. The RBD–SpyTag and HBsAg–Spy-Catcher VLPs were each purified separately and then conjugated through a Good Manufacturing Process (GMP) to produce the RBD–VLP antigen. The conjugation process comprised adding the RBD SARS CoV2 Spy-Tag to the HBsAg VLP Spy-Catcher in a 1:1 proportion and incubating at 2 to 8 °C for 48 to 96 hrs. The purified RBD SARS CoV-2 HBsAg VLP conjugate subunit as a potential vaccine candidate was characterised for its structural and functional properties by liquid chromatography hyphenated with high-resolution mass spectrometry (LC-MS), CD spectroscopy, and fluorescence spectroscopy.

ACE2–hFc protein (ACE2 ectodomain fused to human Fc) was expressed transiently in Expi293F cells (Gibco, Thermo Fisher Scientific, Waltham, MA, USA). Briefly, Expi293F cells were grown as a suspension in Expi293F expression medium at 37 °C, 125 rpm, and 8% CO_2_. At a density of 3 × 10^6^ cells/mL, the cells were transfected with ACE2–hFc plasmid using an Expifectamine293 transfection kit according to the manufacturer’s protocol. The supernatant was harvested 5 days post-transfection and bound to Protein G Sepharose 4 Fast flow resin (GE Healthcare, Uppsala, Sweden). A ten-column volume wash with 1× PBS (pH 7.4) was given, and the protein was eluted with 0.1 M Glycine (pH 2.5) and neutralised by 1 M Tris (pH 9.0). The protein was quantified using the NanoDrop 2000c instrument (Thermo Fisher Scientific, Waltham, MA, USA) by measuring absorbance at 280 nm and using the theoretical extinction coefficient.

Hemagglutinin (HA) is the major surface glycoprotein of influenza virus. mMH1-02TE and mMH3-02TE are His-tagged HA ectodomains from the H1 and H3 influenza subtypes, respectively. These proteins were purified from mammalian Expi293F cell culture supernatants, as described previously [40].

### 2.3. Thermal Stress Studies

The thermal stabilities of the examined proteins were evaluated by nanoDSF (Prometheus NT.48, Nano Temper Technologies, GmbH, Munich, Germany), as described previously [41]. Protein samples were heated from 20 to 95 °C at a ramp rate of 1 °C/min, and the apparent thermal melting temperature (*T*_m_) was determined by monitoring the changes in the fluorescence ratio (*F*_350_/*F*_330_) as a function of temperature.

RBD and HBsAg–RBD conjugate proteins were premixed with CpG1018 adjuvant at a final CpG1018 concentration of 3 mg/mL. The protein–CpG1018 mixing was carried out overnight at 4° C on a rotomixer.

Aliquots of RBD proteins, with and without CpG1018, were incubated at 37 °C at different time points (prolonged thermal stress) and different temperatures (37, 40, 50, 70, and 90 °C) for 1 h (1 h thermal stress) prior to *T*_m_ measurements.

RBD, RNaseA, and HA ectodomain proteins were premixed with CpG1018 or CpG 7909 adjuvants at different weight ratios of protein–CpG. The mixing was carried out overnight at 4 °C on a rotomixer, and the thermal stability, *T*_m_, was examined by nanoDSF.

### 2.4. SPR Binding Studies

Binding studies of proteins to ACE2–hFc were performed on a ProteOn XPR36 Protein Interaction Array V.3.1 (Bio-Rad, Hercules, CA, USA). Initially, the activation of the GLM sensor chip was performed using sulfo-NHS and EDC (Sigma-Aldrich, Darmstadt, Germany). The activation was followed by the immobilisation of (10 μg/mL) Protein G (Sigma-Aldrich, Darmstadt, Germany) in a buffer containing 10 mM sodium acetate, pH 4.0, at a flow rate of 30 μL/min for 300 s up to ~3000 RU. Finally, the quenching of the remaining activated, un-immobilised sulfo-NHS esters was performed using 1 M ethanolamine. ACE2–hFc ligand capture was carried out up to ~500 RU at a flow rate of 30 μL/min for 100 s on the desired channels, excluding a single blank channel that was used as a reference. RBD and the tested protein analytes were passed over the chip at varying concentrations, ranging from 6.25 nM to 100 nM, at a flow rate of 30 μL/min for 200 s for association and 600 s for dissociation. A blank analyte lane was used for passing 1X PBST buffer for referencing. Regeneration was carried out after each kinetic assay using 0.1 M Glycine-HCl, pH 2.5, and the ligand capture was repeated before starting the next kinetic assay. The data were fitted to a simple 1:1 Langmuir interaction model using Proteon Manager to obtain *K_on_*, *K_off_*, and *K_D_* values. Post-thermal stress treatment samples were passed on the chip at a fixed concentration of 100 nM for all samples. The data were fitted to the Langmuir 1:1 binding model.

### 2.5. NanoDSF Studies

The thermal unfolding of RBD, HBsAg–RBD conjugate, RNaseA, and HA ectodomain proteins in the presence or absence of CpG1018 or CpG7909 was carried out by nanoDSF using a Prometheus NT.48 instrument. The assays were carried out with a 1 mg/mL concentration of each protein, and the apparent thermal stability (*T*_m_) was determined by monitoring the changes in the fluorescence ratio (*F*_350_/*F*_330_) as a function of temperature. Samples were heated from 20 to 95 °C with a ramp rate of 1 °C/min.

### 2.6. Proteolysis Studies

Proteolysis assays were carried out using TPCK-Trypsin at 37 °C. Protein substrates were dialysed in MQ water and reconstituted in the digestion buffer (50 mM Tris, pH 7.5, 1 mM CaCl_2_). The proteins were incubated with or without CpG overnight at 4 °C before proteolysis with TPCK-trypsin at a ratio of 1:50 (TPCK Trypsin–protein). Proteolysis was carried out at 37 °C, except for mMH1-02TE and mMH3-02TE proteins, where trypsin digestion was carried out at 4 °C due to the rapid digestion of these proteins by trypsin at 37 °C.

Equal aliquots of samples were taken at 0, 1, 5, 10, 20, 30, and 60 min, respectively. The proteolysis reaction was quenched by adding SDS-PAGE reducing dye, followed by incubation at 95 °C for 10 min and then analysis by SDS-PAGE. CpG was added at a fixed concentration of 3 mg/mL for all proteins. Protein aliquots stored at 4 °C without trypsin were used as controls and termed as time point 0.

### 2.7. Thermal Unfolding as a Function of pH

A concentrated stock of RBD protein was diluted to a final concentration of 0.5 mg/mL. The dilutions were carried out in a series of pH-varying CGH-10 buffers (10 mM each of Citrate, Glycine, and HEPES), with a pH ranging from 4.0 to 10.0 (1 pH unit interval). The samples, alone or mixed with CpG1018 at a final concentration of 3 mg/mL, were incubated for 30 min at room temperature, and then the apparent thermal stability (*T*_m_) was determined by monitoring the changes in the fluorescence ratio (*F*_350_/*F*_330_) as a function of temperature.

### 2.8. Thermal Unfolding as a Function of Salt Concentration

RBD proteins were diluted to a final concentration of 0.5 mg/mL. The dilutions were carried out in a series of Molarity-varying CGH buffers at pH 7.4 (buffer molarity ranges from 10 mM to 1.0 M of Citrate, Glycine, and HEPES). The samples, alone or mixed with CpG1018 at a final concentration of 3 mg/mL, were incubated for 30 min at room temperature, and then the apparent thermal stability (*T*_m_) was determined by monitoring the changes in the fluorescence ratio (*F*_350_/*F*_330_) as a function of temperature.

### 2.9. Thermal Reversibility

RBD proteins were used at a 0.5 mg/mL concentration, alone or mixed with CpG1018 at a final concentration of 3 mg/mL. The thermal unfolding of RBD proteins in the presence or absence of CpG1018 was carried out by nanoDSF using a Prometheus NT.48 instrument, and the apparent thermal stability (*T*_m_) was determined by monitoring the changes in the fluorescence ratio (*F*_350_/*F*_330_) as a function of temperature. In the first cycle, samples were heated from 20 to 70 °C with a ramp rate of 1 °C/min. Once cooled down, the samples were subjected to a second cycle of thermal unfolding and heated from 20 to 70 °C with a ramp rate of 1 °C/min.

### 2.10. Statistical Analysis

For the nanoDSF measurements, each experiment was carried out in duplicate (n = 2), unless mentioned otherwise. All the other experiments were carried out in biological replicates (n = 3). Data analysis was performed using GraphPad Prism software version 8.0.

## 3. Results

### 3.1. CpG Alters Protein Thermal Stability with No Observed Effect on Ligand Binding

**Figure 1:**
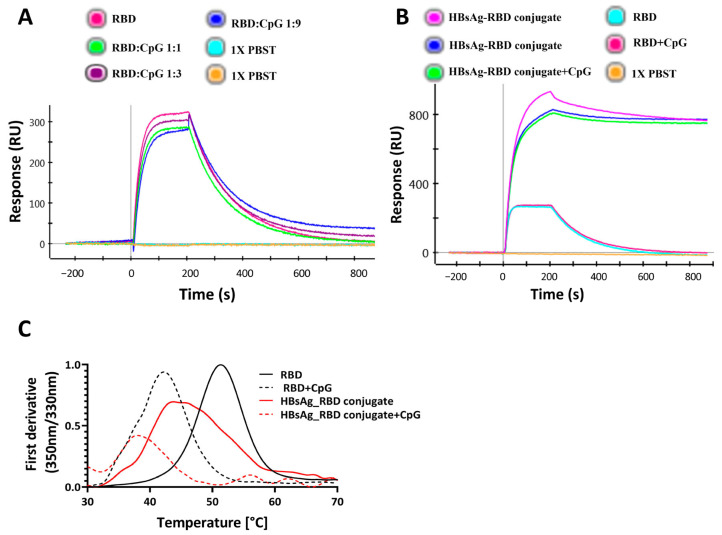
Effect of CpG1018 on the stability and binding of RBD derivatives to ACE2–hFc. (**A**) The effect of CpG1018 on the binding of RBD protein, at 100 nM concentration, to the ACE2–hFc receptor in the presence or absence of CpG1018 adjuvant was performed using SPR. RBD and CpG were mixed at different weight ratios as indicated in 1X PBST buffer, pH 7.4. (**B**) RBD and HBsAg–RBD conjugate were mixed with CpG1018 at a weight ratio of 1:3 protein to CpG. The binding of proteins to the captured ACE2–hFc was carried out using SPR at a uniform concentration of all proteins (100 nM). (**C**) Thermal denaturation profiles of RBD and HBsAg–RBD conjugate proteins with and without CpG1018. CpG was added at a concentration of 3 mg/mL. The intensity values of the first derivative of the fluorescence intensity ratio (350 nm/330 nm) are plotted [42]. The experiments were performed in triplicate.

To evaluate SARS-CoV-2 spike RBD protein stability and binding to its cognate receptor, ACE2, in the presence of a CpG class B TLR9 agonist adjuvant, CpG1018 was incubated with SARS-CoV-2 RBD protein overnight, and the binding of RBD to ACE2 and its thermal denaturation profile were assessed using SPR and nanoDSF, respectively. The SPR results indicated comparable binding profiles for RBD alone and RBD mixed with CpG to ACE 2 (Figure 1A).

Also, for the HBsAg–RBD conjugate, ACE2 binding was not affected in the presence of CpG1018 (Figure 1B). In the presence of CpG, a shift in *T*_m_ towards lower temperatures was observed for both RBD and the HBsAg VLP–RBD conjugate (Figure 1C).

We extended the study to another class B adjuvant, CpG7909, to investigate whether the observed effect was limited to CpG1018 or could be extended to other CpGs. We studied the effects of different concentrations of these CpGs on protein thermal stability.

**Figure 2:**
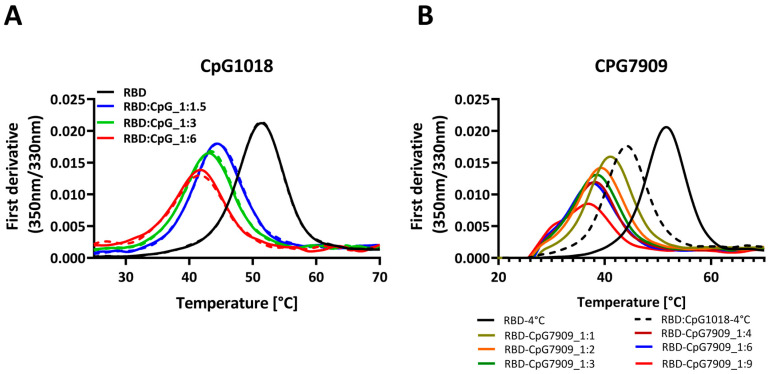
Thermal denaturation profiles of RBD in the presence and absence of two different CpG adjuvants. (**A**) Thermal denaturation profile of RBD alone or mixed with increasing concentrations of CpG1018, ranging from 1.5 to 6 mg/mL per 1 mg/mL of RBD. Each sample was tested in duplicate, as indicated by solid and dashed lines. RBD protein was used at 0.3 mg/mL concentration in 1X PBST buffer, pH 7.4. (**B**) Thermal denaturation profile of RBD alone or mixed with increasing concentrations of CpG7909, ranging from 1 to 9 mg/mL per 1 mg/mL of RBD. RBD protein was used at 0.3 mg/mL concentration in 1× PBST buffer, pH 7.4. Samples mixing with CpG adjuvants were performed in varying weight ratios ranging from 1:1 to 1:9 of RBD to CpG.

When either CpG, CpG1018, or CpG7909 were added at different concentrations to RBD, the destabilising effect of CpG on RBD was observed to be concentration dependent, with a decrease in the protein *T*_m_ as the adjuvant concentration increased. At a CpG concentration of 6 mg/mL, the RBD’s *T*_m_ was lowered by as much as 10 °C (Figure 2A,B). With an increasing CpG concentration, the fluorescence intensity of the peak was also reduced (Figure S1). Overall, these results suggest that, for RBD, CpG had a negative effect on protein thermal stability, with no observed effect on protein binding, as seen in Figure 1B,C. This is suggestive of an enhanced interaction of CpG with the denatured protein, relative to the native state of RBD.

### 3.2. CpG Increased Protein Sensitivity to Proteolytic Cleavage

**Figure 3:**
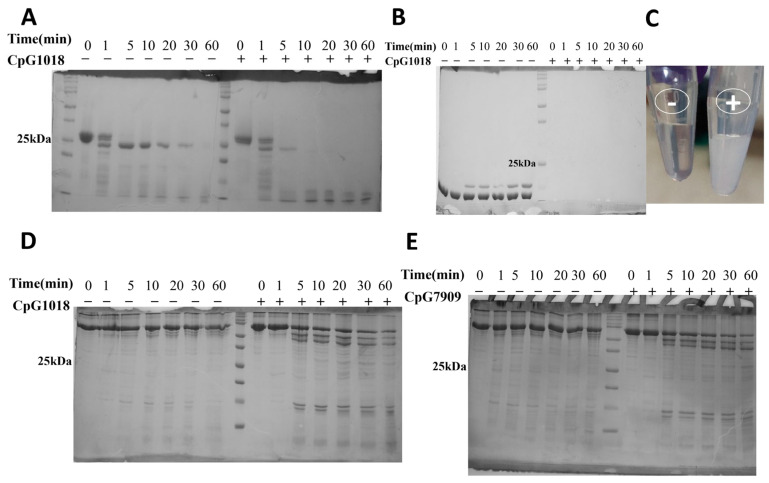
Proteolytic cleavage of proteins by TPCK-treated trypsin. Protein samples were dialysed in MQ water, reconstituted in the digestion buffer (50 mM Tris, (pH 7.5), 1 mM CaCl_2_), and then incubated with or without CpG overnight at 4 °C prior to proteolysis with TPCK-trypsin. Proteolysis was carried out at a ratio of 1:50 (TPCK Trypsin:protein) at 37 °C. Equal aliquots of samples were taken at various time points and the reaction was quenched by the addition of reducing SDS dye and boiling at 95 °C for 10 min. Samples were subsequently analysed by SDS PAGE. (**A**) Trypsin digestion of RBD. (**B**) Trypsin digestion of lysozyme. (**C**) The precipitation of lysozyme upon addition of CpG (tube on the right) compared to a clear solution without CpG (tube on the left). (**D**,**E**) Trypsin digestion of BSA in the presence/absence of (**D**) CpG1018 and (**E**) CpG7909. The original SDS gel figures can be found in Supplementary File S1.

To investigate the effect of CpG on protein proteolytic stability, we subjected different proteins (RBD, lysozyme, and BSA) to proteolytic digestion by TPCK-treated trypsin at different time points in the presence and absence of two class B CpG adjuvants, namely CpG1018 and CpG7909. The results showed that SARS-CoV-2 RBD displayed a good resistance to proteolytic cleavage when digested with trypsin in the absence of CpG1018 (Figure 3A). The addition of CpG1018 increased protein sensitivity to proteolytic cleavage, and this was the case not only for SARS-CoV-2 RBD, but also for lysozyme and BSA (Bovine serum albumin) (Figure 3B–D). In the case of lysozyme, an immediate effect was observed. As the lysozyme came into contact with CpG, it precipitated, and a turbid solution was formed compared to a clear solution in the case of lysozyme without CpG (Figure 3C). After pelleting down, removing the precipitated lysozyme, and loading the clear supernatant on an SDS PAGE gel, no protein band was visible. This indicates that all the lysozyme was precipitated upon the addition of CpG.

A similar enhancement in trypsin cleavage was observed for BSA with CpG1018 and CpG7909, indicating that the CpG effect is not limited to CpG1018, but likely can be generalised to class B CpGs (Figure 3D,E). The same results were observed when the RBD and BSA proteins were dialysed directly against 50 mM Tris buffer, pH 7.5, instead of water, and CaCl_2_ was then added to a final concentration of 1 mM, followed by trypsin digestion at 25 °C (Figure S2A) or 37 °C (Figure S2B,C).

### 3.3. The Destabilising Effect of CpG on Protein: Ligand Binding Is Enhanced with Prolonged Incubation

**Figure 4:**
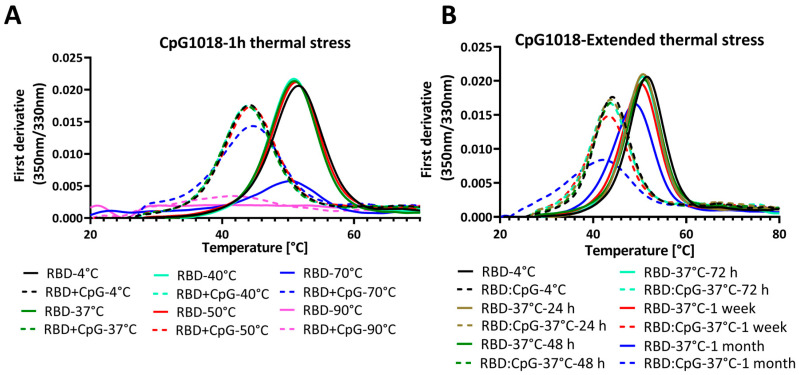
Thermal unfolding profiles of RBD in the presence and absence of CpG1018 adjuvant. The RBD protein was used at a concentration of 0.3 mg/mL in 1X PBS buffer, pH 7.4. (**A**) Thermal unfolding profile of RBD alone or mixed with CpG1018 at a concentration of 3 mg/mL, and then subjected to incubation at different temperatures ranging from 4 °C to 90 °C for 1 h. (**B**) Thermal unfolding of RBD alone or mixed with CpG1018 at a concentration of 3 mg/mL and then subjected to incubation at 37 °C as a function of time.

To assess the effect of CpG on proteins at different temperatures, samples of RBD with and without CpG were incubated at different temperatures for 1 h. The samples were cooled, and *T*_m_ was subsequently assessed using nanoDSF. RBD samples without adjuvants were thermally stable and had a similar *T*_m_ after one hour of incubation at temperatures up to 50 °C. The same was observed for RBD with CpG samples, where the *T*_m_ was stable up to 50 °C, even though it was lower than that of RBD without CpG. After one hour of incubation at 70 °C, the *T*_m_ did not change, but the fluorescence intensity decreased. After incubation at 90 °C, there were no clear transitions in both samples, with and without CpG (Figure 4A). Since the *T*_m_ of RBD was ~50 °C, the data suggest that CpG does not alter folding reversibility, except at incubation temperatures of 70 °C and above.

We next performed an extended thermal stress study, where RBD was incubated with CpG1018 at a 3 mg/mL concentration for different time points up to 1 month at 37 °C. The thermal denaturation profile and binding to ACE2 were studied by nanoDSF and SPR, respectively.

Interestingly, the nanoDSF results showed that RBD without CpG was stable at 37 °C for up to 1 week, and after that, the *T*_m_ started to decrease and a decrease in fluorescence intensity peak was observed (Figure 4B). Conversely, RBD with CpG samples had a lower *T*_m_ compared with that of RBD without CpG. The difference in *T*_m_ between the RBD and RBD with CpG samples was almost constant for time points up to 1 week, and the *T*_m_ started to decrease after that. Also, the decrease in fluorescence intensity at the thermal transition peak was higher for RBD with CpG at the 1 week and 1 month time points (Figure 4B).

**Figure 5:**
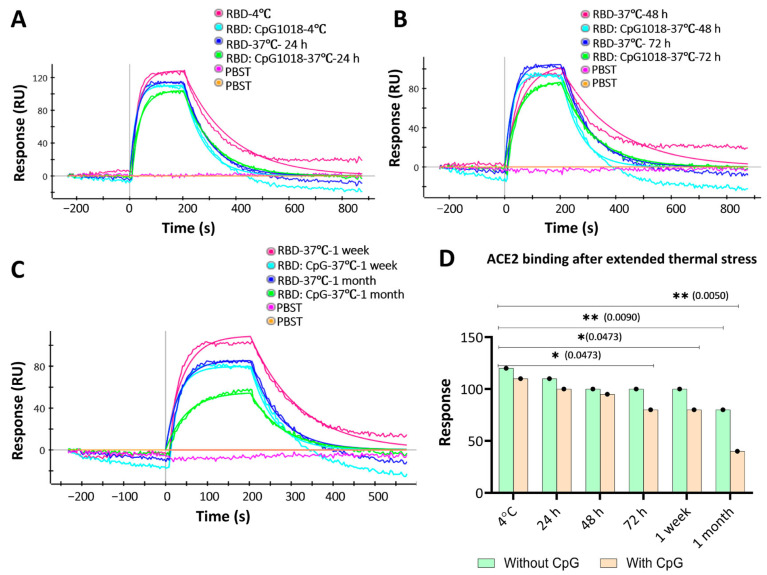
ACE2 binding to RBD with and without CpG1018 after incubation at 37 °C for varying times. Aliquots of RBD with and without CpG were kept at 4 °C as a control. PBST was used as a negative control to establish a baseline. RBD protein was used at a fixed concentration (100 nM). (**A**) Binding profile of samples stored at 4 °C and 37 °C for 24 h. (**B**) Binding profile of samples stored at 37 °C for 48 and 72 h. (**C**) Binding profile of samples stored at 37 °C for 1 week and 1 month. (**D**) Bar graph summarising the RU response to ACE2 binding for the samples studied after thermal stress. Significance levels are indicated as: * *p* < 0.05; ** *p* < 0.01, and the comparisons that were not significant are not shown.

Consistent with the nanoDSF results, the SPR results showed a similar binding profile to ACE2 for samples incubated with and without adjuvants at 37 °C for up to 1 week (Figure 5A–C). At further time points, the binding response started to decrease. Interestingly, the RBD with CpG samples showed a more significant drop in binding response after 1 week than the RBD without CpG samples, and the binding response dropped even further at 1 month of incubation time relative to control samples stored at 4 °C (Figure 5C). These results indicate that CpG has no effect on protein binding characteristics at short times, but this effect is prominent at incubation times of a week or more (Figure 5D).

### 3.4. The Destabilising Effect of CpG Is Not Limited to RBD

**Figure 6:**
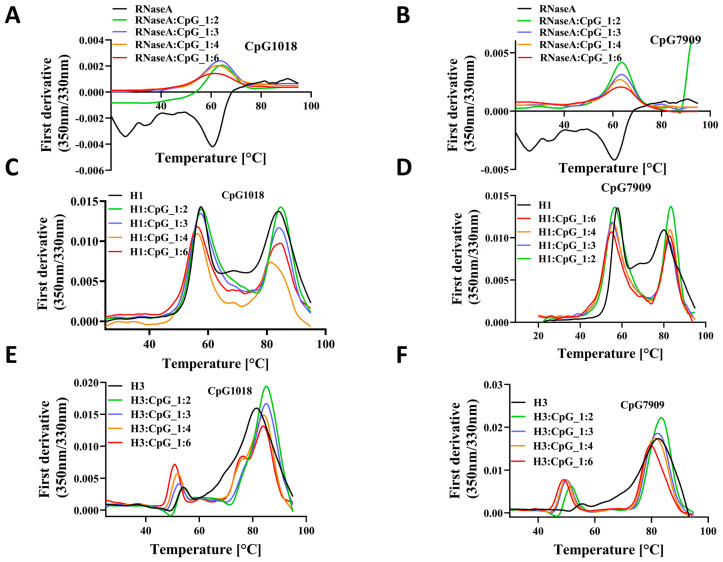
The effect of CpG on the thermal stability of RNaseA and HA proteins. Thermal denaturation profile of RNaseA as a function of either (**A**) CpG1018 or (**B**) CpG7909 concentrations. Thermal denaturation profiles of influenza H1-HA ectodomain as a function of concentration of (**C**) CpG1018 or (**D**) CpG7909. Thermal denaturation profiles of influenza H3-HA ectodomain as a function of concentration of (**E**) CpG1018 or (**F**) CpG7909.

Different proteins other than RBD were used to extend the previous findings. We studied the thermal stability of RNaseA, lysozyme, and Influenza virus Hemagglutinin (HA) ectodomain proteins upon mixing with CpG1018 and CpG7909. In the case of RNaseA, there was no significant shift in *T*_m_ with varying concentrations of CpG, but there was a decrease in the fluorescence intensity ratio with increasing concentrations of both CpG1018 and CpG7909 (Figure 6A,B). Lysozyme completely precipitated upon adding the CpG, and no clear transition was observed in *T*_m_. The ectodomain of HA of the group 1 influenza (H1-HA) ectodomain protein exhibited a decrease in fluorescence peak intensity with increasing concentrations of CpG, and the *T*_m_ was lowered by about 5 °C for both CpG1018 and CpG7909 (Figure 6C,D). A similar *T*_m_ shift was observed for another influenza group 2 ectodomain protein (H3-HA), but without a decrease in fluorescence intensity. Both the H1 and H3 ectodomain proteins showed a second transition at higher temperatures, which indicates protein aggregates. Interestingly, the aggregates seemed to be resistant to CpG destabilisation (Figure 6C–F).

We next examined the proteolytic sensitivity profiles for the ectodomains of H3-HA and SARS-CoV-2 spike, since they are both vaccine candidates for influenza and COVID-19, respectively [43,44,45]. Thermal stability analysis of the spike was not performed, as it shows multiple transitions and denaturation is not reversible [46]. TPCK-trypsin digestion was performed. A minimum or no effect of CpG on protein proteolytic sensitivity was observed, which differs from the CpG effect observed on RBD, BSA and lysozyme (Figure S3A,B).

### 3.5. The Effect of CpG on RBD Thermal Stability Is pH-Independent and Salt-Concentration-Dependent

**Figure 7:**
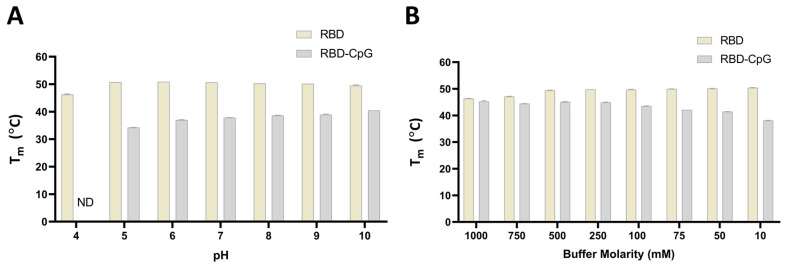
The effect of pH and salt on the thermal stability of RBD with and without CpG. *T*_m_ was measured using nano-DSF by monitoring the changes in the fluorescence ratio (*F*_350_/*F*_330_) as a function of temperature. (**A**) *T*_m_ of RBD at a concentration of 0.5 mg/mL in CGH-10 buffer containing 3 mg/mL CpG1018 as a function of pH. (**B**) *T*_m_ of RBD with and without 3 mg/mL CpG as a function of salt concentration. All experiments were performed in duplicate. ND: not determined.

We assessed the effect of CpG on RBD protein’s thermal stability in buffers at different pHs ranging from 4 to 10 (Figure 7 and Figure S4). RBD protein displayed similar thermal unfolding profiles and a similar *T*_m_ throughout the pH range from 5.0 to 10.0, while a slight shift to a lower *T*_m_ was observed in the buffer with a pH of 4.0. When CpG1018 was added to RBD, no thermal transition was obtained in the pH 4.0 buffer, while at other pHs, a shift in *T*_m_ from 10 to 20 °C lower than the T_m_ of RBD alone was observed. A pH-dependent shift in *T*_m_ was observed when CpG1018 was added to RBD in buffers with a pH range between 5.0 and 10.0, with an increase in *T*_m_ as the buffer pH increased (Figure 7A and Figure S4). In the highest pH buffer, *T*_m_ was still ~10 °C lower than that of the RBD protein without CpG.

The effect of salt concentration on the binding of CpG to RBD protein was also assessed by incubating RBD with and without CpG in buffers with varying salt concentrations ranging from 10 mM to 1.0 M. RBD protein displayed similar thermal unfolding profiles and a similar *T*_m_ in the salt concentration ranges from 10 mM to 500 mM, while a shift to a lower *T*_m_ was observed at salt concentrations higher than 500 mM. When CpG1018 was added to RBD, no changes in *T*_m_ at high salt concentrations (750 mM and 1.0 M) were seen. However, a shift to a lower *T*_m_ was observed when CpG was added to RBD in buffers with salt concentrations lower than 750 mM. We noticed a salt-concentration-dependent shift in *T*_m_ when CpG1018 was added to RBD in the salt concentration range from 10 mM to 750 mM, with an increase in *T*_m_ as the buffer salt concentration increased (Figure 7B). At the highest molarity buffer, the *T*_m_ was similar to that of the RBD protein without CpG in the same buffer. RBD thermal unfolding and *T*_m_ were the same in all buffers with salt concentrations ranging between 10 mM and 500 mM.

When assessed by repetitive cycles of thermal unfolding, a highly reversible thermal unfolding profile was observed for RBD with and without CpG (Figure S5).

## 4. Discussion

Adjuvants are critical components of protein subunit vaccine formulations. They contribute to enhancing immune responses both in terms of quality and duration, especially in the case of protein subunit vaccines. A modest humoral immune response is elicited by protein vaccines in the absence of adjuvants, with minimum to no T-cell responses, and this requires administering multiple immunisations to achieve a good vaccine efficacy. Adjuvants are added to amplify humoral and cellular responses to reduce the number of immunisations and enable the use of lower vaccine doses [47]. However, as adjuvants are biologically and chemically active components that could impact the molecular structure and/or stability of a vaccine antigen, formulation work is often needed to develop a stable formulation for an adjuvanted vaccine candidate, particularly for a single-vial or pre-filled vaccine formulation. CpG adjuvants are TLR9 agonists that trigger proinflammatory responses such as the secretion of IL-12 and IFNs and induce potent Th1 immune responses with Cytotoxic T Lymphocyte (CTL) activity [48,49]. Recently, significant advancements have been made in the field of adjuvant and vaccine formulation development, focused on enhancing their safety, efficacy, and durability, but less effort has been directed toward studying the antigen stability and conformation in these formulations. A previous study reported that CpG adjuvants affected the thermal stability of HIV trimer derivatives by about +2.0 °C for BG505 and −2.0 °C for B41. The same study found that CpG adjuvants bind to the cationic region of the HIV envelop apex, occluding the PGT145 bNAb epitope [50], however, the impact on in vivo immunogenicity was not evaluated. Another recent study reported decreased stability, represented by a >10 °C decrease in the *T*_m_ of a monomeric RBD antigen in the presence of CpG [51]. In the same study, a ~30% loss of native RBD protein was observed when formulated with Alhydrogel + CpG (AH–CpG) and stored at 4 °C, at a rate of ~10% loss per month [51]. The AH–CpG formulation, which was unstable in vitro, was more immunogenic than RBD formulated solely with either AH or CpG in vivo, suggesting that in vitro readouts in these studies do not necessarily predict in vivo immunogenicity. However, in the same study, the authors observed that the formulation stored at 37 °C for a week prior to immunisation elicited significantly lower neutralisation titres after a single immunisation than the corresponding material stored at 4 °C. After two immunisations, this difference disappeared. In the present study, we investigated the effect of one of the commonly used CpG adjuvants on the stability of different proteins and vaccine candidates. RBD protein expressed in mammalian cells [46] or *Pichia pastoris* (Serum Institute of India) and HBsAg–RBD conjugate with RBD expressed in *Pichia pastoris* were formulated with different concentrations of two class B CpG adjuvants. The results indicated a negative impact of CpG on the thermal stability of these proteins. The proteins’ *T_m_* decrease was concentration-dependent, and at higher CpG concentrations, the *T_m_* dropped by > 10 °C. Even though CpG affected the thermal stability of these proteins, their binding to the ACE2 receptor was not altered when monitored by SPR after short-term incubation. Interestingly, the effect of CpG on protein binding manifested upon prolonged incubation and caused a drastic decrease in the fraction of functional protein. When studied by nanoDSF, it was also observed that protein fluorescence was quenched upon CpG formulation in a concentration-dependent manner. To further investigate the stability of the protein in the presence of CpG, proteolytic cleavage studies were performed, and they clearly showed a significant increase in the proteolytic sensitivity of different proteins upon CpG addition. The immediate destabilising effect of CpG was clearly visible when added to lysozyme, as it caused an instant aggregation of the lysozyme. The mechanism of the destabilising effect of CpG adjuvants was not clear, but one possibility is the direct association of CpG through its negative phosphate groups to positive patches present in the protein, followed by aggregation driven, in part, by base stacking between CpG ODNs bound to different protein molecules, as in the case of lysozyme. Since proteins are positively charged at pHs lower than their pI, we expected a significant decrease in *T*_m_ due to an increase in binding to CpG at lower pHs, at least in the case of RBD, which has a calculated pI of 8.6. However, when RBD was incubated with CpG at varying pHs, no significant pattern was observed for the decrease in *T*_m_ as a function of pH from pH 5.0 to 10.0. The decrease in thermal stability observed for several proteins was likely because of the preferential interaction of CpG with the denatured rather than the native state. The fluorescence reduction in the presence of CpG was likely because of collisional quenching by CpG in the native state of the protein.

When this study was extended to different proteins, the CpG effect was inconsistent. Some proteins exhibited significant conformational destabilisation displayed by a decreased thermal and proteolytic stability, while other proteins were only slightly affected and others were unaffected. It is not clear why CpG adjuvants are destabilising for some proteins but not for others. Still, this is a significant concern for vaccine development and emphasises the importance of protein conformational and stability characterisation in the presence of these adjuvants. While adjuvants might not impact immunogenicity and conformational stability after short-term storage, there might be effects with long-term storage, especially at elevated temperatures. While no major effect of pH on the CpG-mediated destabilisation of RBD was observed, CpG destabilisation increased with decreasing salt concentrations. An increase in salt concentration shielded the protein-charged residues from interacting with CpG, but there is a limit as to what level the salt concentration can be increased without negatively affecting protein stability. Similar studies need to be performed with other adjuvants as well. We previously examined the effect of extended storage of RBD derivatives [40] in SWE-adjuvanted preparations and found that the formulations retained their antigenicity and other biophysical characteristics, even after month-long storage at 37 °C [40]. These studies highlight the need to investigate the impacts of other adjuvants used in different vaccine formulations on vaccine stability after prolonged storage prior to immunisation. This is especially true for vaccine deployment in LMICs, where cold chain maintenance can be challenging. Protein conformational stability can have a direct effect on B-cell epitope availability. Protein destabilisation can lead to the loss of conformational epitopes, resulting from an enhanced fraction of protein in the unfolded state. Antigen destabilisation by CpG may promote the local unfolding of some regions in the protein, which may compromise the integrity of the neutralising epitopes.

There are some limitations of this work. While our work suggests the need for in vitro studies for the development of suitable, stable formulations of vaccine candidates, particularly when an adjuvant is considered, only biophysical characterisation was carried out in this work, without any immunogenicity studies. We have previously shown [51] that small enhancements in protein thermal stability can significantly enhance neutralising antibody titres. CpG adjuvants have been used successfully for multiple licensed commercial vaccines, including HEPLISAV-B and five different COVID vaccines. Therefore, additional studies exploring multiple doses and storage conditions will be required to understand if and how in vitro characterisation, such as that presented in this study, correlates with the in vivo immunogenicity of a CpG-adjuvanted vaccine. Another limitation is that the study is limited to one type of adjuvant, CpG, and only one class of CpG. It remains to be further investigated if other non-CpG commercial adjuvants have similar in vitro characteristics in such studies. Additionally, while our study focuses on the biophysical characterisation of the CpG effect on a few proteins, the understanding of the molecular mechanism behind this destabilisation is still lacking, and biophysical studies such as HDX-MS could be useful to elucidate the nature of CpG interaction with proteins and the associated effects on protein stability.

## 5. Conclusions

The present work demonstrates that class B CpG adjuvants can impact protein stability, likely by stabilising the unfolded state. Their effects are protein-specific, and the molecular mechanisms remain to be elucidated. This highlights the importance of careful and systematic in vitro formulation studies for the development of suitable, stable formulations of adjuvanted vaccine candidates.

## Data Availability

The data relevant to the figures in the paper have been made available within the article and in the Supplementary Information section. All unique/stable reagents generated in this study are available from the Lead Contact Raghavan Varadarajan (varadar@iisc.ac.in).

## Supplementary Materials

The following supporting information can be downloaded at: https://www.mdpi.com/article/10.3390/vaccines13040395/s1, Figure S1: Effect of CpG1018 on the protein fluorescence count; Figure S2: Trypsin digestion of proteins dialysed against Tris buffer pH 7.5 and then CaCl_2_ was added to a final concentration of 1 mM; Figure S3: The effect of CpG on the proteolytic sensitivity of different proteins; Figure S4: The effect of pH on the thermal stability of RBD with and without CpG; Figure S5: Thermal Reversibility profile of RBD with and without CpG1018; File S1: The original SDS gel figures.
