# Supplementary material for: Destabilising Effect of Class B CpG Adjuvants on Different Proteins and Vaccine Candidates
Source: Vaccines (Basel). 2025 Apr 8;13(4):395. doi: 10.3390/vaccines13040395 (PMC12031019; doi:10.3390/vaccines13040395)
Supplement: Supplementary file 1 [file vaccines-13-00395-s001.zip › File S1. The original SDS gel figures.pdf]

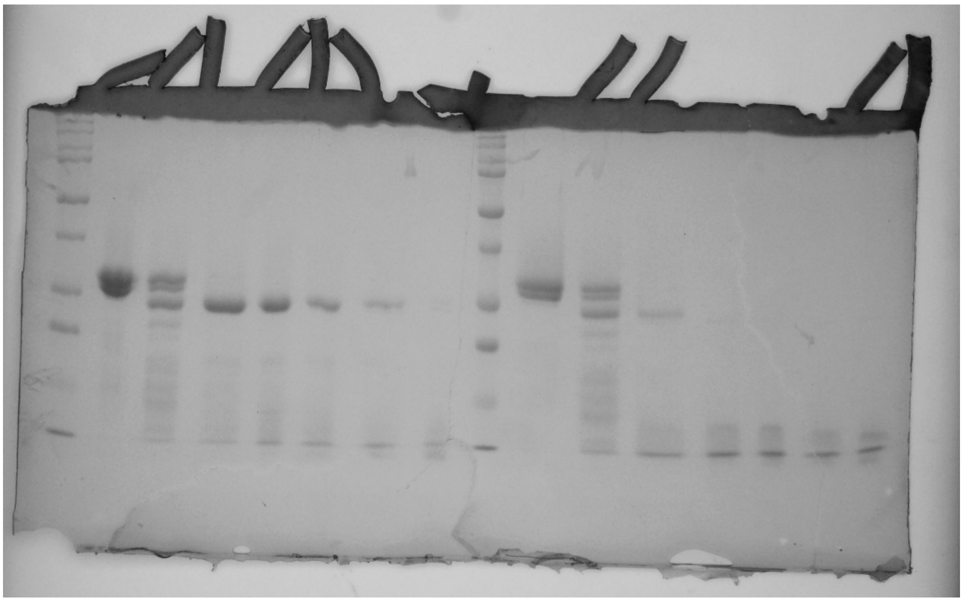

Figure 3A: Proteolytic cleavage of RBD protein by TPCK-treated trypsin.

Lane1 and lane 9: protein marker

Lane 2 to lane 8 are for RBD protein without CpG1018 incubated with TPCK trypsin for time points: 0, 1, 5, 10, 20, 30, and 60 min, respectively.

Lane 10 to lane 16 are for RBD protein with CpG1018 incubated with TPCK trypsin for time points: 0, 1, 5, 10, 20, 30, and 60 min, respectively.

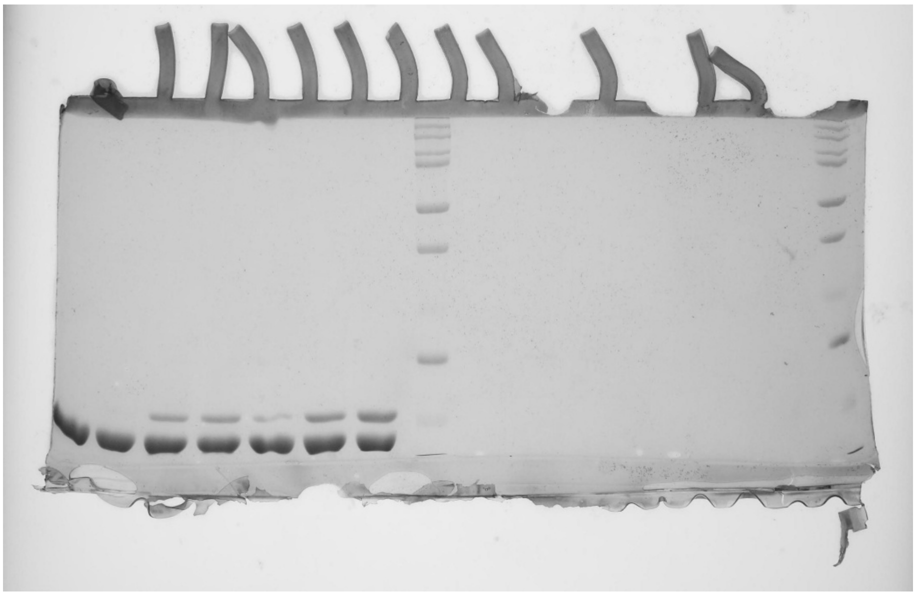

Figure 3B: Proteolytic cleavage of Lysozyme protein by TPCK-treated trypsin.

Lane 8 and lane 16: protein marker

Lane 1 to lane 7 are for Lysozyme protein without CpG1018 incubated with TPCK trypsin for time points: 0, 1, 5, 10, 20, 30, and 60 min, respectively.

Lane 9 to lane 15 are for Lysozyme protein with CpG1018 incubated with TPCK trypsin for time points: 0, 1, 5, 10, 20, 30, and 60 min, respectively.

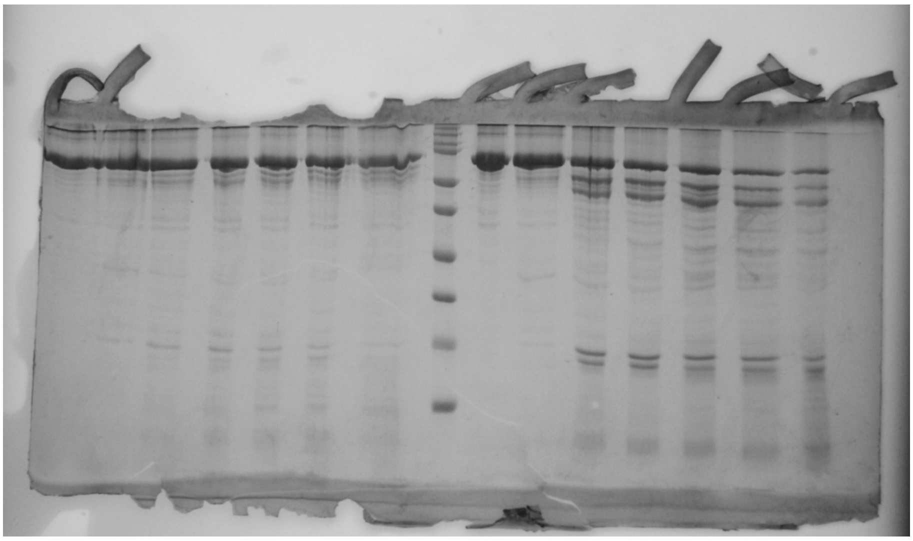

Figure 3D: Proteolytic cleavage of BSA protein by TPCK-treated trypsin.

Lane 8 : protein marker

Lane 1 to lane 7 are for BSA protein without CpG1018 incubated with TPCK trypsin for time points: 0, 1, 5, 10, 20, 30, and 60 min, respectively.

Lane 9 to lane 15 are for BSA protein with CpG1018 incubated with TPCK trypsin for time points: 0, 1, 5, 10, 20, 30, and 60 min, respectively.

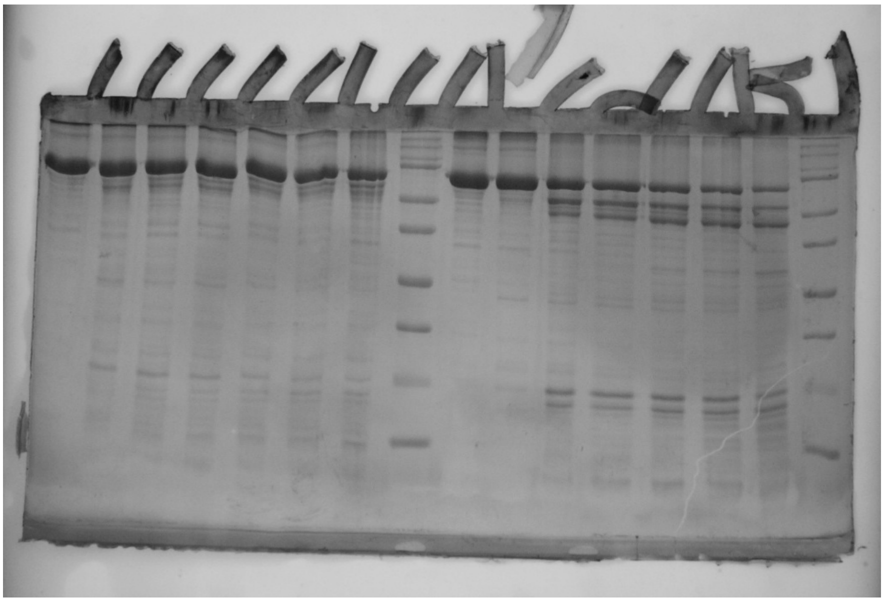

Figure 3E: Proteolytic cleavage of BSA protein by TPCK-treated trypsin.

Lane 8 and lane 16: protein marker

Lane 1 to lane 7 are for BSA protein without CpG7909 incubated with TPCK trypsin for time points: 0, 1, 5, 10, 20, 30, and 60 min, respectively.

Lane 9 to lane 15 are for BSA protein with CpG7909 incubated with TPCK trypsin for time points: 0, 1, 5, 10, 20, 30, and 60 min, respectively.

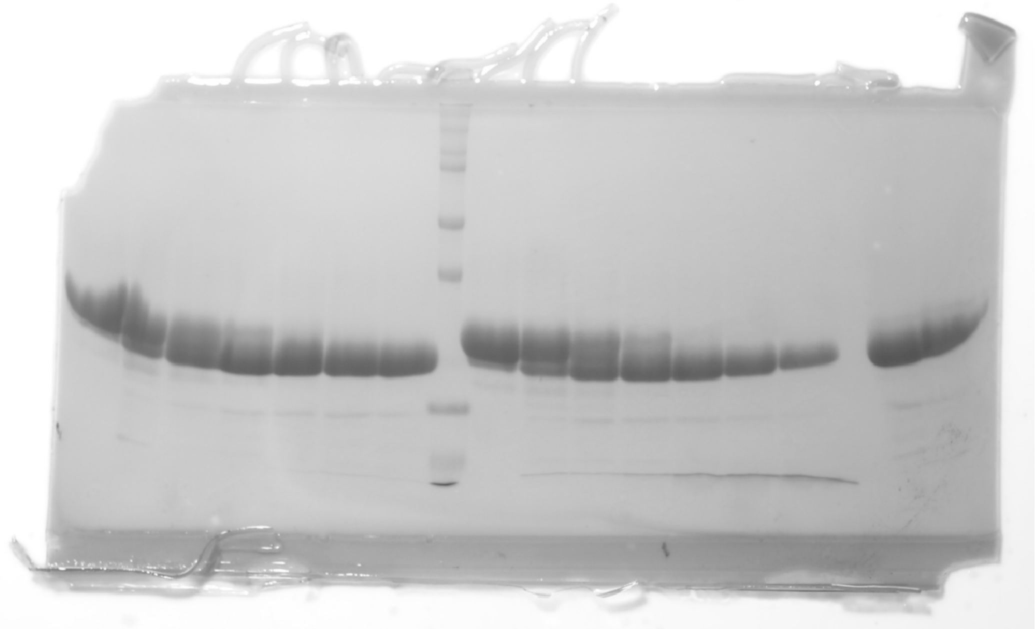

Figure S2A: Trypsin digestion of RBD protein at 25°C. The protein was dialysed against Tris buffer pH 7.5 and then CaCl2 was added to a final concentration of 1 mM.

Lane 8: protein marker

Lane 1 to lane 7 are for RBD protein without CpG1018 incubated with TPCK trypsin for time points: 0, 1, 5, 10, 20, 30, and 60 min, respectively.

Lane 9 to lane 15 are for RBD protein with CpG1018 incubated with TPCK trypsin for time points: 0, 1, 5, 10, 20, 30, and 60 min, respectively.

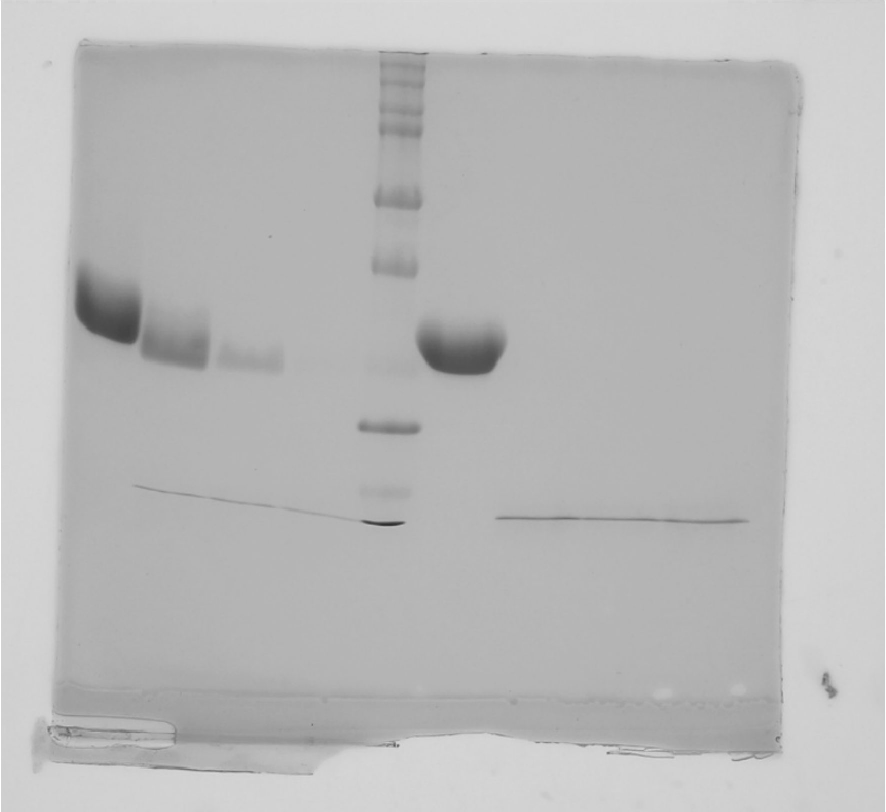

Figure S2B: Trypsin digestion of RBD protein at 37°C. The protein was dialysed against Tris buffer pH 7.5 and then $CaCl_2$ was added to a final concentration of 1 mM.

Lane 5: protein marker

Lane 1 to lane 4 are for RBD protein without CpG1018 incubated with TPCK trypsin for time points: 0, 10, 30, and 60 min, respectively.

Lane 6 to lane 9 are for RBD protein with CpG1018 incubated with TPCK trypsin for time points: 0, 10, 30, and 60 min, respectively.

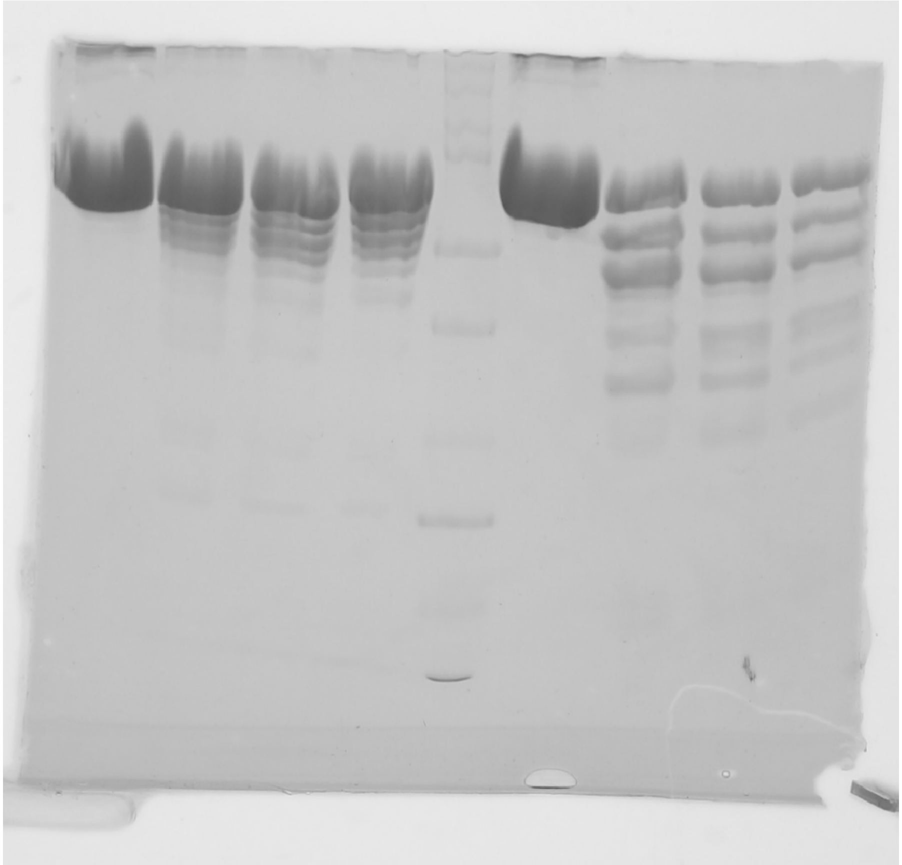

Figure S2C: Trypsin digestion of BSA protein at 37°C. The protein was dialysed against Tris buffer pH 7.5 and then $CaCl_2$ was added to a final concentration of 1 mM.

Lane 5: protein marker

Lane 1 to lane 4 are for BSA protein without CpG1018 incubated with TPCK trypsin for time points: 0, 10, 30, and 60 min, respectively.

Lane 6 to lane 9 are for BSA protein with CpG1018 incubated with TPCK trypsin for time points: 0, 10, 30, and 60 min, respectively.

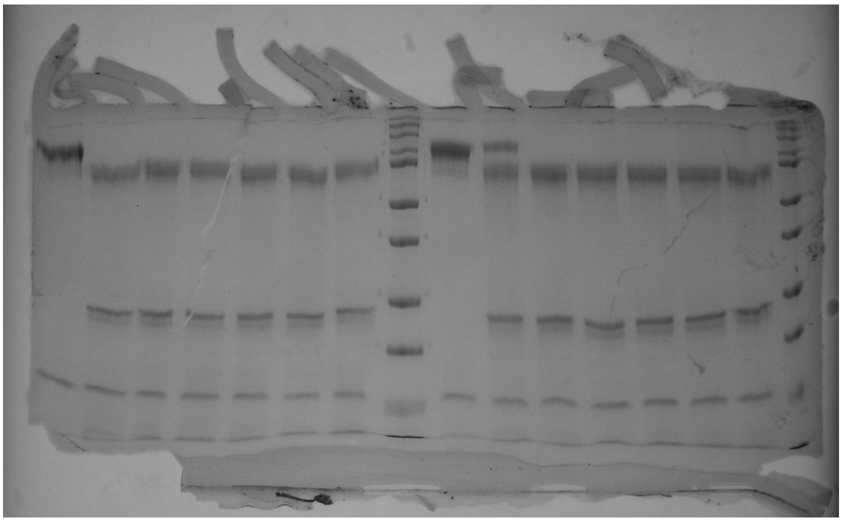

Figure S3A: Proteolytic cleavage of H3-HA ectodomain protein by TPCK-treated trypsin.

Lane 8 and lane 16: protein marker

Lane 1 to lane 7 are for H3-HA ectodomain protein without CpG1018 incubated with TPCK trypsin for time points: 0, 1, 5, 10, 20, 30, and 60 min, respectively.

Lane 9 to lane 15 are for H3-HA ectodomain protein with CpG1018 incubated with TPCK trypsin for time points: 0, 1, 5, 10, 20, 30, and 60 min, respectively.

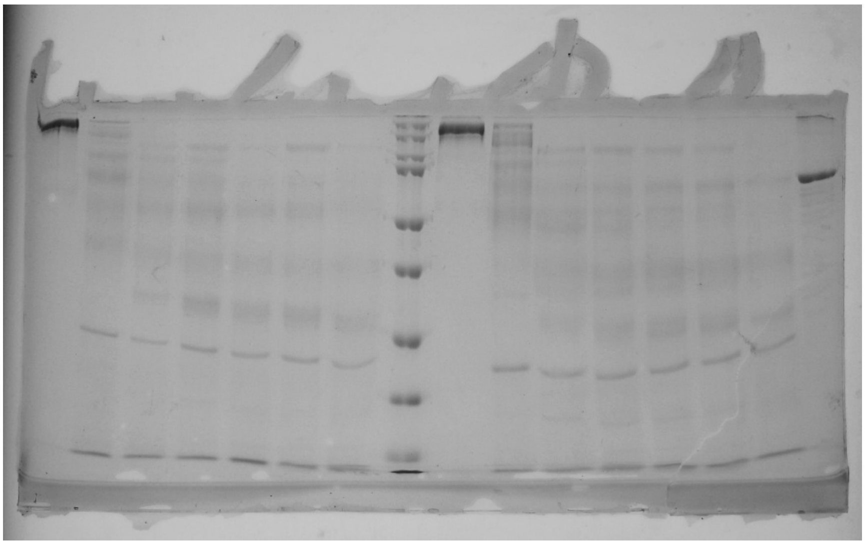

Figure S3B: Proteolytic cleavage of SARS-CoV-2 Spike protein by TPCK-treated trypsin.

Lane 8: protein marker

Lane 1 to lane 7 are for SARS-CoV-2 Spike protein without CpG1018 incubated with TPCK trypsin for time points: 0, 1, 5, 10, 20, 30, and 60 min, respectively.

Lane 9 to lane 15 are for SARS-CoV-2 Spike protein with CpG1018 incubated with TPCK trypsin for time points: 0, 1, 5, 10, 20, 30, and 60 min, respectively.
