# Supplementary material for: Destabilising Effect of Class B CpG Adjuvants on Different Proteins and Vaccine Candidates
Source: Vaccines (Basel). 2025 Apr 8;13(4):395. doi: 10.3390/vaccines13040395 (PMC12031019; doi:10.3390/vaccines13040395)
Supplement: Supplementary file 1 [file vaccines-13-00395-s001.zip › vaccines-3426321-Supplementary.pdf]

Destabilising Effect of Class B CpG Adjuvants on Different Proteins and Vaccine Candidates

Kawkab Kanjo [1], Rakesh Lothe [2], Gaurav Nagar [2], Meghraj Rajurkar [2], Harish Rao [2], Saurabh Batwal [2], Umesh Shaligram [2] and Raghavan Varadarajan [1,*]

[1] Molecular Biophysics Unit, Indian Institute of Science, Bangalore 560012, India

[2] Serum Institute of India Pvt. Ltd., Pune 411028, India

* Correspondence: varadar@iisc.ac.in, Tel.: +91-80-22932612; Fax: +91-80-23600535

Supporting figures

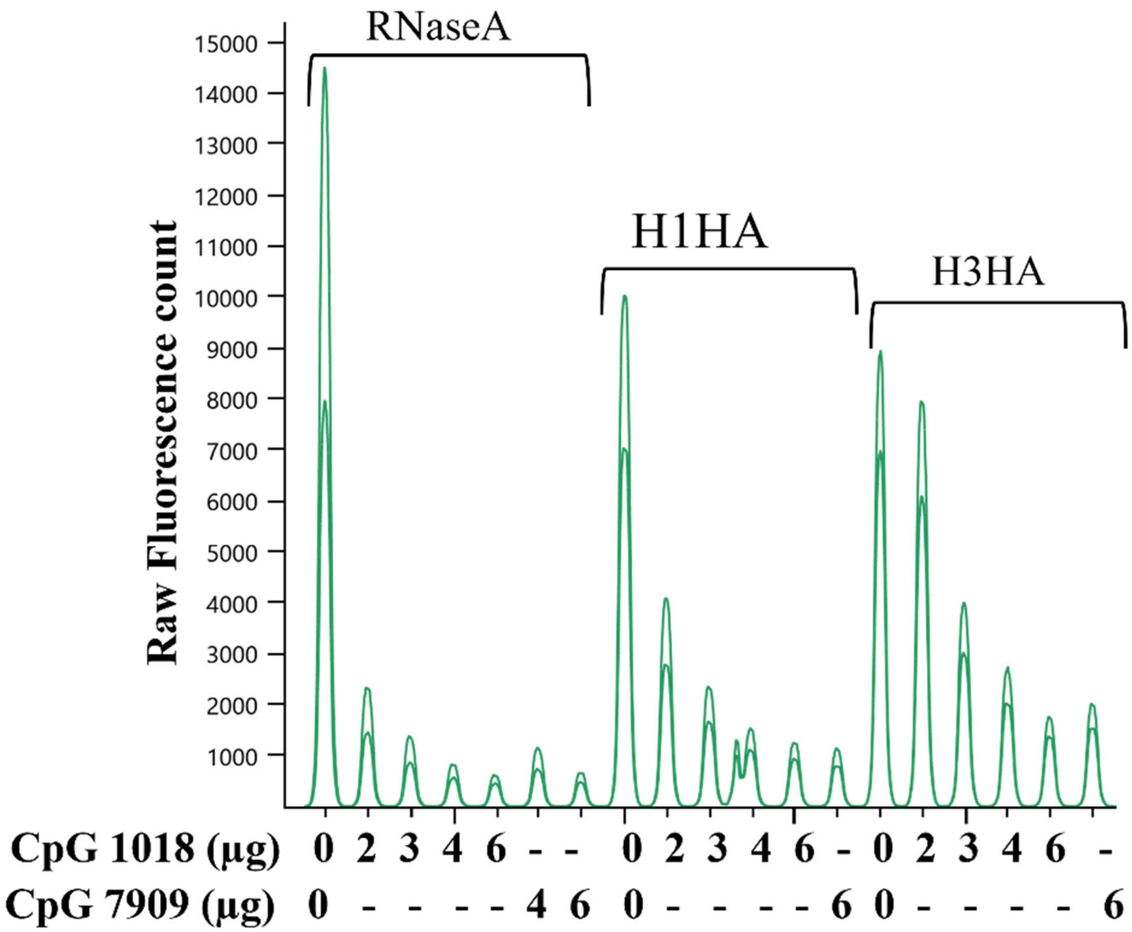

Figure S1. Effect of CpG1018 on the protein fluorescence count. The fluorescence count was carried out prior to the nanoDSF experiment, where 0.3 mg/ml of each protein sample was mixed with varying amounts of CpG adjuvants in 1X PBST, pH 7.4. The amount used of each CpG adjuvant is indicated below the figure in μg of adjuvant used per 1 μg of protein. With increasing CpG concentration, the fluorescence intensity is reduced, possibly because of fluorescence quenching by CpG. The fluorescence count for each sample is measured at 330 and 350 nm wavelengths.

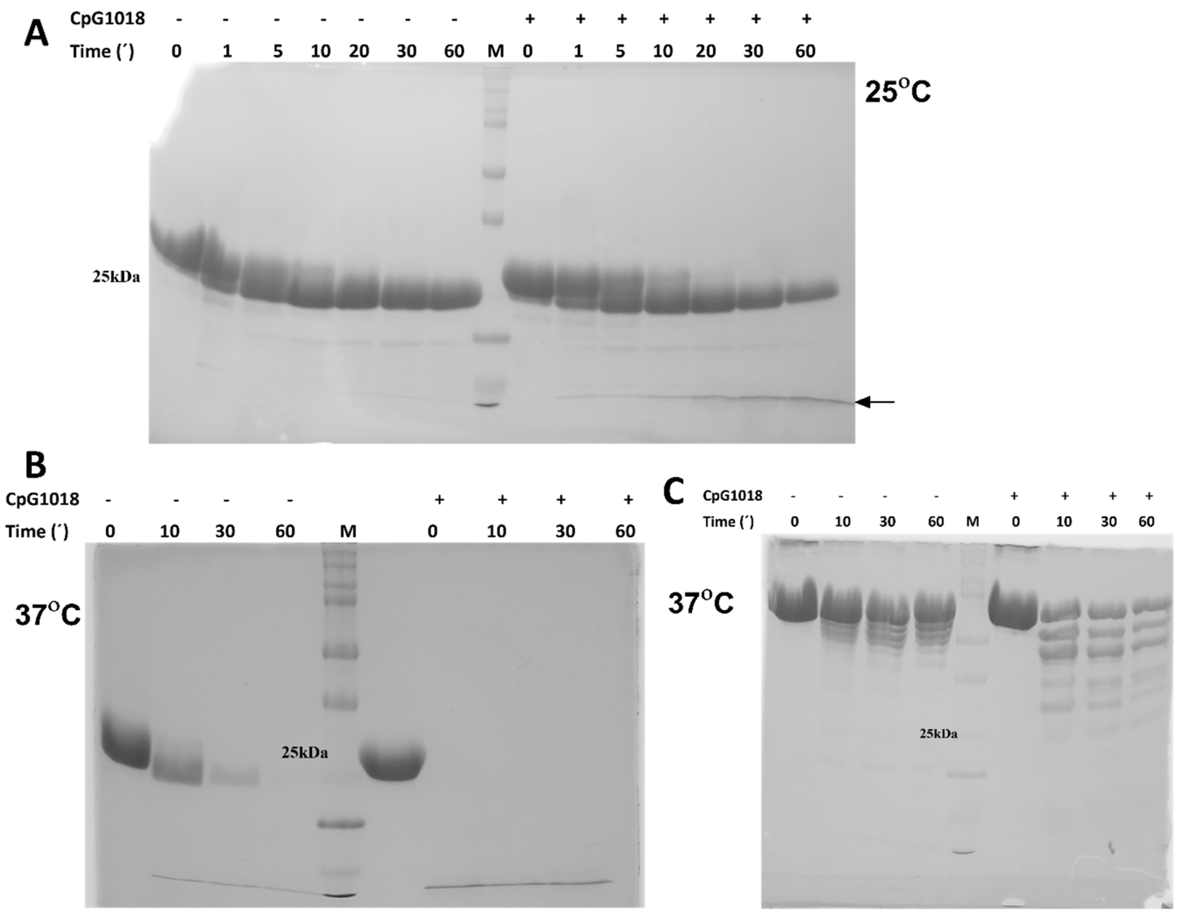

Figure S2. Trypsin digestion of proteins dialysed against Tris buffer pH 7.5 and then CaCl2 was added to a final concentration of 1 mM. (A) RBD protein digestion at 25°C. (B) RBD protein digestion at 37°C. (C) BSA protein digestion at 37°C.

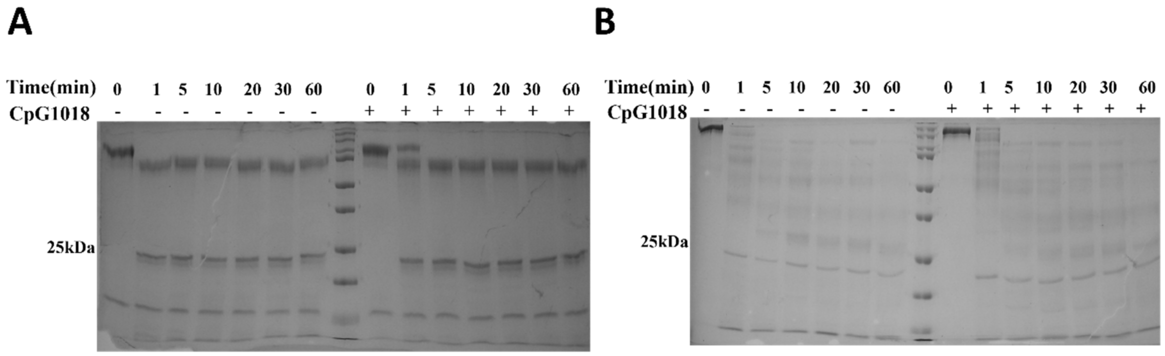

Figure S3. The effect of CpG on the proteolytic sensitivity of different proteins. Protein samples were dialyzed in MQ water and reconstituted in the digestion buffer (50 mM Tris, pH 7.5, 1 mM $CaCl_2$ and then incubated with or without CpG overnight, at 4°C prior to proteolysis with TPCK-trypsin at a ratio of 1:50 (TPCK Trypsin: protein). Proteolysis was carried out at 4°C, due to the fast digestion of these proteins by trypsin at 37°C. Equal aliquots of samples were taken at various time points and the reaction was quenched by the addition of reducing SDS dye and boiling at 95°C. Samples were subsequently analysed by SDS PAGE (A) Trypsin digestion of the H3-HA ectodomain. (B) Trypsin digestion of SARS-CoV-2 Spike.

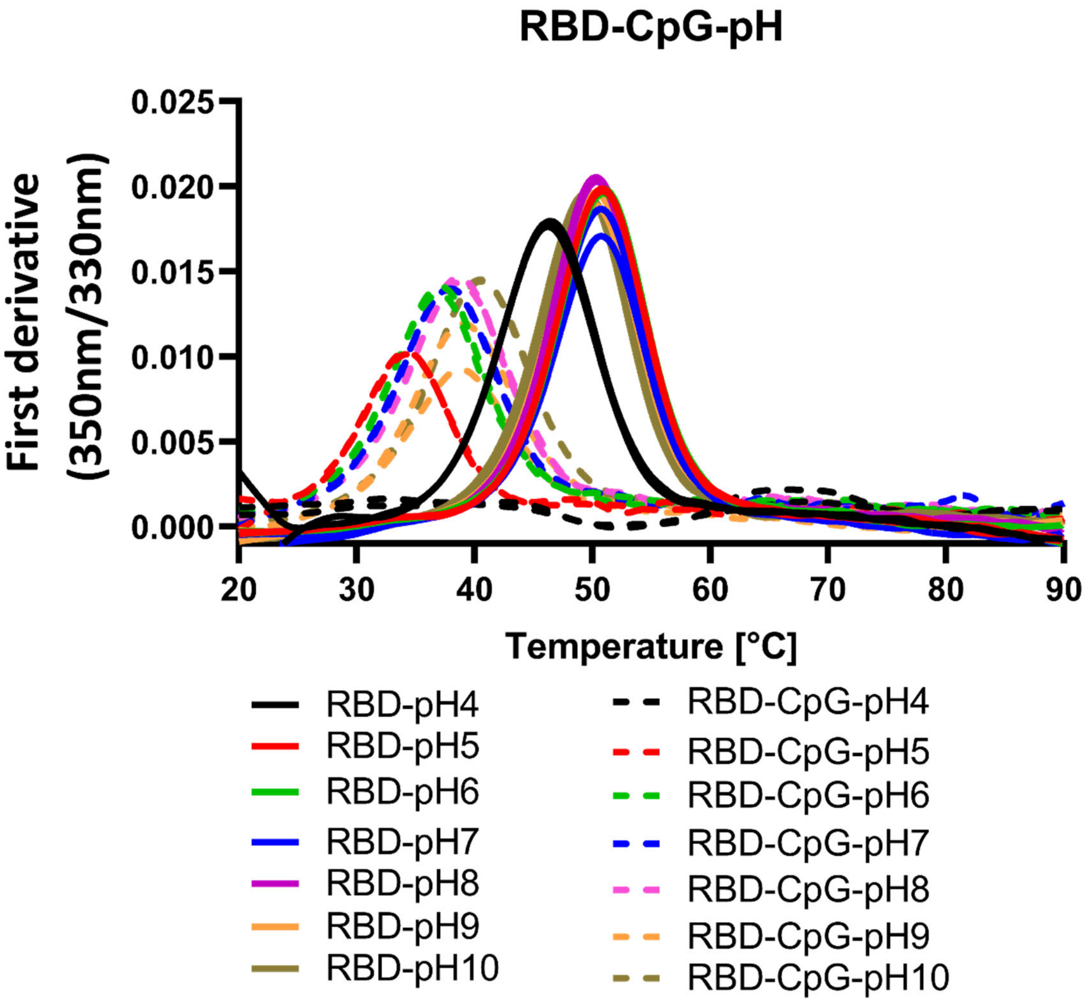

Figure S4. The effect of pH on the thermal stability of RBD with and without CpG. A concentrated stock of RBD protein was diluted to a final concentration of 0.5 mg/ml in CGH-10 buffer with different pH (4.0, 5.0, 6.0, 7.0, 8.0, 9.0, 10) and formulated with CpG1018 to a final concentration of 3 mg/ml. The thermal unfolding profile of RBD with and without CpG1018 (solid and dashed lines, respectively) was evaluated, and the ratio of the first derivative of *F350* nm/*F330* nm was plotted as a function of temperature. The experiment was done in duplicate.

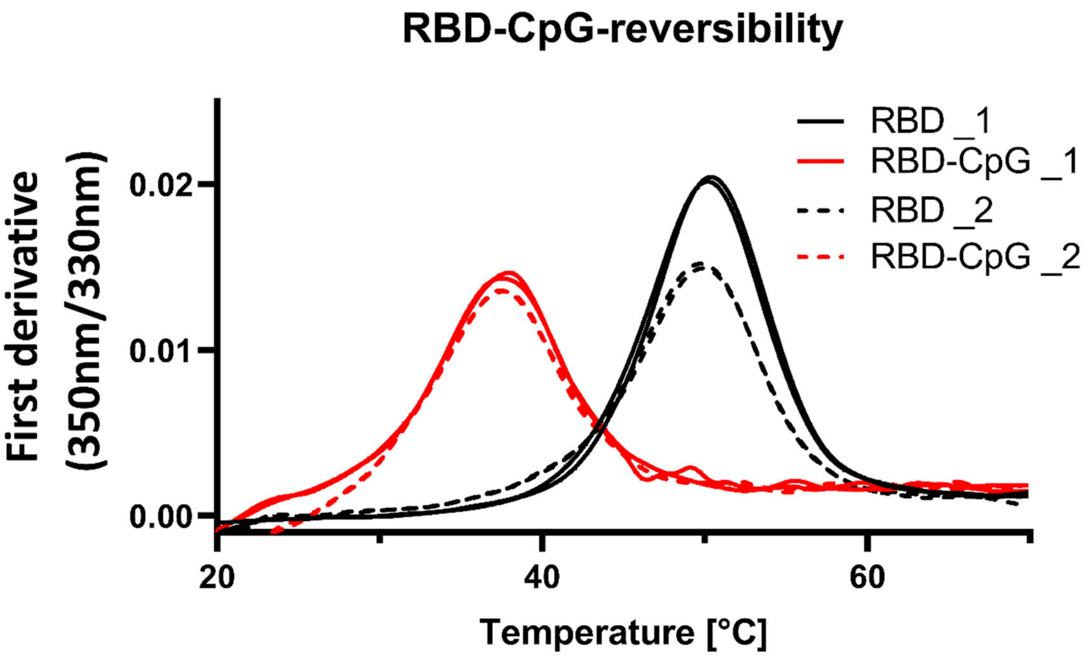

Figure S5. Thermal Reversibility profile of RBD with and without CpG1018. RBD protein in buffer (50 mM Tris, 1 mM CaCl2 (pH 7.5) was incubated with or without CpG overnight, at 4°C and then subjected to the first cycle (RBD_1 and RBD-CpG_1) of thermal unfolding from 20 to 70°C. The samples were cooled down and subjected again to a second cycle (RBD_2 and RBD-CpG_2) of thermal unfolding from 20 to 70°C.
